# Comparisons of Thermo-Oxidative Ageing Performance and Lifespan Evaluation of Grafted Polypropylene and XLPE Cables: Combined Effect of Temperature and Thickness

**DOI:** 10.3390/polym18030386

**Published:** 2026-01-31

**Authors:** Wenjia Zhang, Shangshi Huang, Mingti Wang, Juan Li, Wei Wang, Shixun Hu, Jinliang He

**Affiliations:** 1 State Key Laboratory of Alternate Electrical Power System with Renewable Energy Sources, North China Electric Power University, Beijing 102206, China; 2 State Key Laboratory of Power System Operation and Control, Tsinghua University, Beijing 100084, China; 3 SINOPEC (Beijing) Research Institute of Chemical Industry Co., Ltd., Beijing 100013, China

**Keywords:** grafted polypropylene, thermo-oxidative ageing, lifespan prediction, recyclable cable

## Abstract

Grafted polypropylene (PPG) has demonstrated significant potential as a recyclable insulation material for high-voltage cables. While its fundamental electrical, mechanical and thermal properties have been widely studied, research on its long-term performance remains insufficient. This study comparatively investigates the thermo-oxidative ageing performance of PPG and traditional cross-linked polyethylene (XLPE) to evaluate the expected lifespan of cable insulation. The evolution of mechanical and electrical properties of PPG and XLPE was monitored during accelerated thermo-oxidative ageing experiments conducted at their respective maximum allowable operating temperatures, and the most sensitive ageing parameter was identified. Furthermore, the influence of thickness on the insulation ageing process was examined through experiments on samples of different thicknesses. Results indicate that the estimated thermo-oxidative ageing lifespan of XLPE at its maximum operating temperatures of 90 °C is 37.75 years, while that of PPG at 110 °C is 45.65 years. This work offers a practical methodology for polymer ageing lifespan analysis and provides valuable insights for assessing the long-term performance of PPG cables in high-voltage applications.

## 1. Introduction

In recent years, the accelerating global industrialization has led to a continuous increase in the demand for electricity. High-voltage cables have gained widespread application due to their remarkable advantages of large transmission capacity and reduced land occupancy [1,2,3,4]. Though cross-linked polyethylene (XLPE), a conventional insulating material for power cables, plays an important role in the safe power transmission [4,5,6,7], it suffers several disadvantages, including a complex manufacturing process and poor recyclability, which not only increase production costs but also pose environmental challenges [1,2,3]. Therefore, there is an urgent need to develop recyclable cable insulation materials to replace XLPE and promote the sustainable development of the cable industry.

Polypropylene (PP), is considered a promising alternative to XLPE due to its excellent electrical insulation strength, convenience of recycling, and lower cost. Therefore, it has attracted extensive research attention [5,6]. To solve the inherent brittleness of PP at low temperatures and meet the growing demands for higher voltage levels and greater transmission capacities, various modification strategies have been explored. By blending PP with various elastomers such as ethylene-propylene rubber [7], ethylene-vinyl acetate copolymer [7], polyolefin elastomers [7,8,9], and hydrogenated styrene−butadiene−styrene block copolymers [9], the flexibility of the modified materials has been improved. However, such blending often compromises electrical insulation properties to varying degrees [7,8,9]. To improve the insulation properties of PP, both nano-doping and grafting modifications have proven effective [5,10]. While PP nanocomposites have demonstrated significant improvements in lab-scale preparations, controlling nanoparticle agglomeration in large-scale industrial production remains challenging, which often results in a reduction in performance [2,3]. In contrast, grafting modification provides more stable improvements to the properties and has become one of the most practically valuable techniques for PP modification [2,3]. Currently, 10 kV, 35 kV, and 110 kV grafting modified PP cables have been successfully applied in China.

Despite the relatively late start of research on grafting-modified PP as a cable insulation material, studies have confirmed its excellent performance in its short-term properties [2,11,12,13,14]. Studies on its long-term durability indicate that grafting monomers such as styrene (St) and methyl methacrylate (MMA) can significantly improve the thermo-oxidative ageing resistance of PP [3,15,16]. However, the expected service life of grafting-modified PP cables remains inconclusive. The conventional lifespan prediction method involves conducting temperature-accelerated ageing experiments to obtain material lifetimes at elevated temperatures, followed by extrapolation to working temperature using the Arrhenius equation [17]. However, the maximum test temperature is limited by the melting temperature (*T_m_*) of the thermo-plastic materials, which restricts the acceleration efficiency. Therefore, this study proposes an innovative ageing methodology that combines temperature and dimensional (thickness) acceleration to enhance the efficiency of lifespan predictions. By comparing the results with those of a widely used 500 kV XLPE material produced by Borealis, a life prediction model is established and applied to predict the life of grafting-modified PP cables. This work aims to provide a conclusion for their expected service life and offer new perspectives for accelerated ageing experiments.

## 2. Materials and Methods

### 2.1. Sample Preparation

The XLPE (Density: 0.92 g/cm^3^, MFR: 2 g/10 min, Crystallinity: 34.38%, T_m_: 102 °C) material selected for this study was Borealis (Vienna, Austria) LE4201S, a 500 kV-grade cable insulation material. The grafting-modified PP cable material, referred to as PPG (Density: 0.94 g/cm^3^, MFR: 2.4 g/10 min, Crystallinity: 43.27%, T_m_: 159 °C) in this paper for brevity, was developed collaboratively by Tsinghua University and the Sinopec Beijing Research Institute of Chemical Industry (Beijing, China) with the brand name 1101. The raw PP (Density: 0.94 g/cm^3^, MFR: 2.4 g/10 min, Crystallinity: 47.53%, T_m_: 164 °C) material used for grafting modification was Sinopec Maoming Petrochemical Company (Maoming, China) T30s, and the styrene (Density: 0.91 g/mL, Mw: 104.15, T_m_: −31 °C) monomer used was S817094 bought from Macklin (Shanghai, China). The used chemical reagents, benzoyl peroxide (BPO, Density: 1.32 g/cm^3^, Mw: 242.23, T_m_: 104 °C) and xylene (Density: 0.86 g/mL, Mw:106.17, T_m_: −34 °C) are B802244 and X820585 respectively, both from Macklin. The synthesis procedure was as follows: PP powder was put into a nitrogen-purged airtight reactor. Then, the initiator BPO mixed with the grafting monomer styrene was added to the PP powder. The mixture was then reacted with xylene solvent at 90 °C for 2–6 h. At last, after filtration, the PP was grafted with St. The more detailed preparation method of PPG is described in the patent 202011195858X [18].

An appropriate amount of XLPE pellets was weighed, and then hot-pressed at 120 °C under 15 MPa for 10 min for shaping. Then, while maintaining the pressure, the temperature was raised to 180 °C and held for 25 min to ensure full crosslinking. Afterwards, the material was water-cooled to 20 °C under 10 MPa for 10 min. Finally, 100, 400, 1000 and 1600 μm sheet samples were yielded. Similarly, an appropriate amount of PPG pellets was weighed, and then hot-pressed at 200 °C under 15 MPa for 10 min, followed by water-cooling under the same conditions, producing sheets of different thicknesses.

To prepare specimens for subsequent Atomic Force Microscopy (AFM) observation, thin-film samples of XLPE and PPG were fabricated via a spin-coating process. First, a 4 wt% solution of each polymer was prepared by dissolving the respective pellets in xylene at 120 °C for 4 h under continuous stirring to ensure complete dissolution. Two gold-plated silicon wafers were then mounted on separate spin coaters. The coaters were set to a temperature of 100 °C, a spin speed of 3000 rpm, and a spin time of 10 s. An appropriate amount of PE and PP solution was, respectively, dropped onto the centre of each silicon wafer, and then, the spin coaters were turned on. After spinning, the wafers were retrieved. Subsequently, the silicon wafer coated with the PE was transferred to a vacuum oven at 180 °C for 4 h to ensure complete crosslinking. Thus, the in situ ageing samples for AFM observation were obtained. 

### 2.2. Accelerated Ageing Experiment Procedure

The XLPE and PPG samples were individually hung in DHG-9070A air ovens under atmospheric conditions, with an air exchange rate of 20 times per hour for accelerated ageing experiments. According to the cable insulation ageing guidelines in GB/T 2951.12-2008, a minimum distance of 20 mm was maintained between vertically suspended samples to ensure uniform heating and oxygen exposure [19]. 

In the temperature-accelerated ageing experiment, sample thickness was standardized to 100 µm to maximize the acceleration effect. Considering the melting point of PPG samples is 159 °C, the maximum test temperature was set to 150 °C. For XLPE, 135 °C is specified as the test temperature according to GB/T 12706.3-2020 [20]. In order to balance fitting accuracy and experimental duration, the final experimental temperatures were selected as 127.5 °C, 135 °C, 142.5 °C, and 150 °C. Based on preliminary tests, appropriate sampling intervals were determined for PPG and XLPE at each temperature. Samples were then taken at these intervals, respectively, for the breakdown performance and mechanical performance tests. To ensure a reliable fitting, a minimum of six valid data points was required.

To enhance the acceleration effect, the test temperature was standardized to 150 °C in the dimensional (thickness) acceleration ageing experiment. Similarly, based on preliminary results, appropriate sampling periods for PPG and XLPE samples of varying thicknesses were determined. Samples were then collected, respectively, at these intervals for the mechanical performance test.

### 2.3. Performance Testing Methods

#### 2.3.1. Thermogravimetry (TG) Testing

The thermal decomposition resistance of the samples can be evaluated using a thermogravimetric analyzer (TA Instruments, TGA Q500, Shanghai, China). Specifically, 5 mg of each sample was placed in a platinum crucible, followed by heating from ambient temperature to 600 °C at a heating rate of 20 °C min^−1^ under a nitrogen atmosphere. The curve depicting the variation in sample mass with temperature was thus obtained. The initial decomposition temperature (*T_onset_*) of the sample was defined as the temperature at which the sample loses 5% of its initial mass.

#### 2.3.2. Conductivity Testing

The volume conductivity of XLPE and PPG samples was tested using a three-electrode system. The testing system is powered by a Keithley (Cleveland, OH, USA) 2290-10 10 kV power supply, and a Keithley 2635B digital source meter is used to collect the leakage current. A DHG-9030A air oven, produced by Beijing Laikaibo Instrument Equipment Co., Ltd., (Beijing, China) is used to provide a specific temperature during the test. Conductivity was measured at room temperature and at the maximum operating temperatures of XLPE (90 °C) and PPG (110 °C).

#### 2.3.3. Tensile Testing

The aged samples were tested to obtain the elastic modulus, tensile strength, and elongation at break using the SANS CMT4304 (Shenzhen, China) universal testing machine. The test continued until the sample fractured. In order to ensure the accuracy and reliability of the test, at least 5 sets of data were collected and averaged for the final data.

#### 2.3.4. Breakdown Strength Testing

The breakdown strength of the aged samples was tested using a Z-VI breakdown tester by Haiwo Science and Technology Co., Ltd. (Jiangsu, China) The spherical electrodes have a diameter of 1.5 cm. Due to equipment and safety constraints, only 100 μm thick samples were tested. During the test, the samples were fully immersed in silicone oil, and the voltage was ramped at 1 kV/s until breakdown occurred. The breakdown voltage was then recorded. To ensure the accuracy and reliability of the results, at least 20 breakdown tests were performed per sample. The characteristic breakdown electric field strength (*E*_0_) was then calculated using the two-parameter Weibull distribution, as shown below:

(1) $$P\left(E\right)=1-\exp\left[-{\left(E/E_{0}\right)}^{\beta }\right]$$

where, *E* is the experimentally measured breakdown strength; *E*_0_ is the scale parameter (characteristic breakdown strength at a cumulative breakdown probability of 63.2%); *β* is the shape parameter, representing data dispersion [21]. The breakdown characteristics were measured at room temperature and at the maximum operating temperatures of XLPE (90 °C) and PPG (110 °C).

#### 2.3.5. FTIR Testing

A Nicolet iS10 FT-IR Spectrometer of Thermo Fisher (Waltham, MA, USA) was used to characterize the chemical structural changes in XLPE and PPG samples before and after ageing. Spectra were acquired for 16 scans with a resolution of 6 cm^−1^ within the range of 4000~652 cm^−1^. Atmospheric background subtraction was carried out, followed by baseline correction and normalization. The reference peak for normalization for XLPE and PPG is both the asymmetric stretching vibration of the methylene group in the range of 2915~2925 cm^−1^.

#### 2.3.6. Scanning Electron Microscopy (SEM)

A Hitachi SU8010 (Tokyo, Japan) was used to observe the microstructural morphology of XLPE and PPG samples before and after ageing. The samples were pre-treated at low temperature by immersing them in liquid nitrogen for 5 min and then cryogenically fractured. The fracture surfaces were mounted on stubs, and uniformly sputter-coated with gold before observation. 

#### 2.3.7. Atomic Force Microscopy (AFM)

The silicon wafers were placed horizontally in two glass Petri dishes with lids (30 mm in diameter) to minimize contamination from airborne dust. The Petri dishes containing the samples were then placed in a DHG-9070A air oven, with the temperature set at 150 °C. The XLPE and PPG samples were taken out periodically, and then, a Bruker Dimension Icon (Karisruhe, Germany) was used to observe the in-situ ageing process. 

## 3. Results and Discussion

### 3.1. Fitting Formula

For subsequent analysis, the performance retention rate is defined as the percentage of a given property (elongation at break, tensile strength, or breakdown strength) retained after ageing time *t*, relative to its initial value:

(2) $$H=C_{t}/C_{0}$$

where, *H* is the retention rate, *C_t_* is the performance at time *t*, and *C*_0_ is the initial performance.

To model the property degradation kinetics, the Dakin model is employed [22]. It describes the rate of change in the concentration of a critical component within the insulation, which is governed by

(3) $$\frac{dC}{dt}=-KC^{n}$$

where, *C* is the concentration of the chemical substance, K is the rate constant, and *n* is the reaction order. It is assumed that the measured physical property P is intrinsically linked to this chemical concentration:

(4) $$P∼C\;\mathrm{or}\;P=f\left(C\right)$$

For a first-order reaction (chain scission in this study), integration of Equation (3) yields an exponential decay of C with time:

(5) $$C=C_{0}e^{-Kt}$$

where, *t* is the reaction time. This implies that the material’s property degrades following a logarithmic relationship with ageing time. More generally, a function of the property can be found that is linear with time:

(6) $$f^{\prime }(P)=-kt$$

Furthermore, combine Equation (6) with the Arrhenius equation:

(7) $$k=A\exp\left(-Ea/RT\right)$$

where, k is the rate constant, A is the frequency factor, *E_a_* is the apparent activation energy, R is the molar gas constant, and *T* is the thermodynamic temperature. A fundamental relationship for the time to reach a specific state of degradation is obtained:

(8) $$\ln\left(t\right)=\frac{E_{a}}{R}\cdot \frac{1}{T}+\ln\left(B\right)$$

where, *t* is the ageing lifetime, with the unit in hours (h). Equation (8) implies a linear relationship between the logarithm of the lifetime and the reciprocal of absolute temperature. This relationship forms the theoretical cornerstone for extrapolating accelerated ageing data obtained at elevated temperatures to predict service life at operational temperatures, as applied in the following sections for XLPE and PPG.

### 3.2. Basic Properties Comparison Between XLPE and PPG

**Figure 1:**
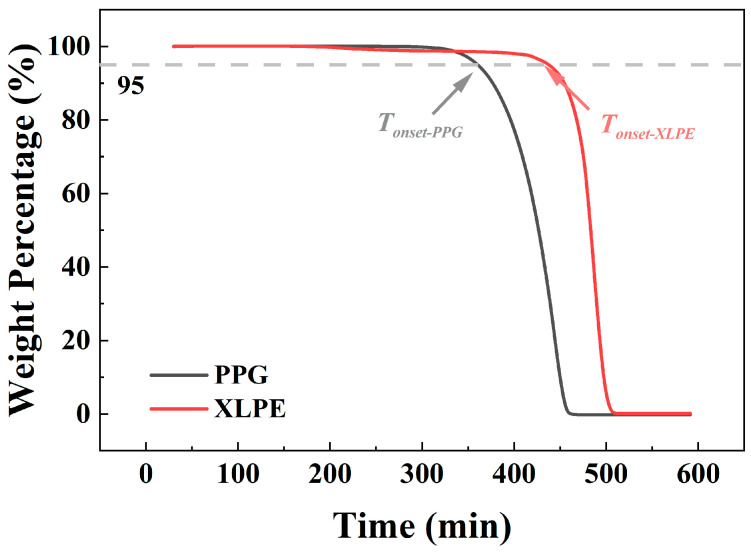
TG curves of XLPE and PPG (dash line: 95% of original weight).

The thermogravimetric (TG) results are presented in Figure 1. In the initial heating stage (0–300 min), both materials exhibit excellent weight retention, maintaining over 95% of their initial mass, indicating good thermal stability in the early phase of elevated temperature exposure. However, a clear divergence in their thermal degradation behavior emerges thereafter: the *T_onset-XLPE_* is 435 °C, whereas *T_onset-PPG_* is 359 °C, revealing that XLPE exhibits superior thermal stability to PPG. Nevertheless, both decomposition temperatures are substantially lower than the maximum operating temperature (typically 90–110 °C for high-voltage cables) and the short-term overload temperature (up to 130 °C) of power cables.

**Figure 2:**
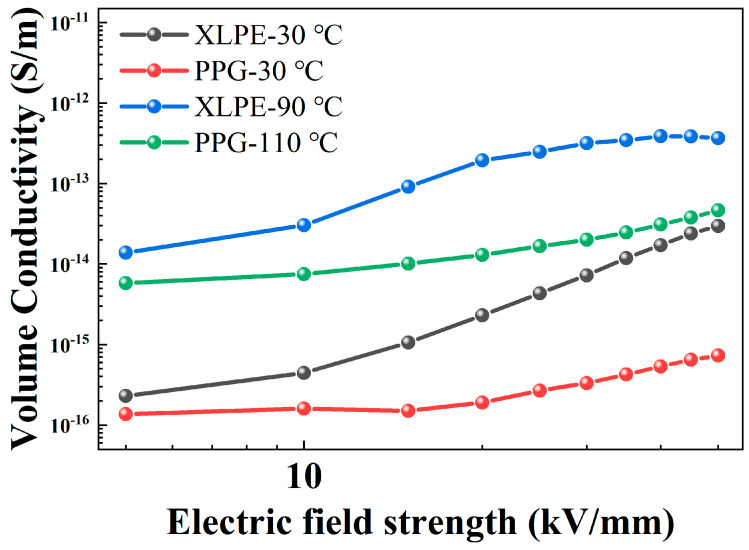
Conductivity of XLPE and PPG under different temperatures.

The volume conductivity results are shown in Figure 2. XLPE has higher conductivity at room temperature. At its operating temperature (90 °C), its conductivity increases rapidly with the electric field. While PPG can maintain lower conductivity characteristics both at room temperature and at its maximum operating temperature of 110 °C, regardless of whether it is under a high electric field or a low electric field, demonstrating its excellent insulating properties.

The initial tensile properties of XLPE and PPG samples with different thicknesses are summarized in Table 1. PPG generally shows higher elastic modulus, elongation at break and tensile strength compared to XLPE across all thicknesses.

**Figure 3:**
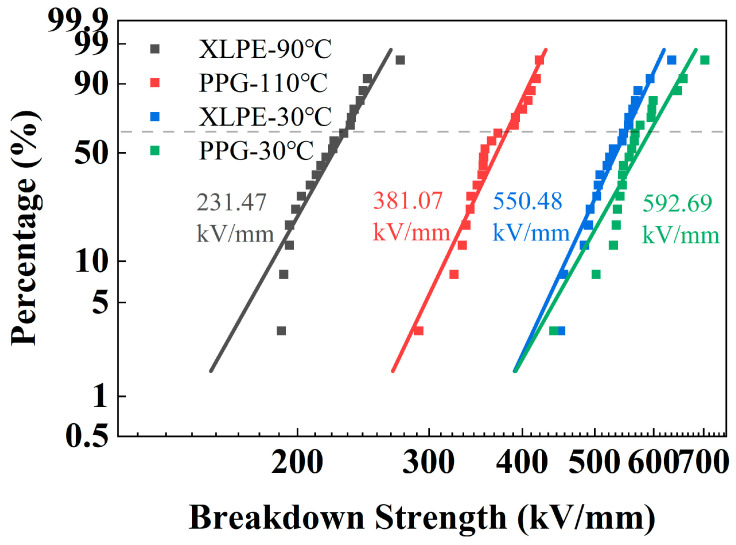
Breakdown results of XLPE and PPG under different temperatures (dash line: 63.2%).

The breakdown results are shown in Figure 3. At room temperature, there is little difference in the breakdown field strength between PPG and XLPE. After the temperature rises to their respective maximum operating temperatures, the breakdown strengths of both PPG and XLPE decrease significantly, but the breakdown strength of PPG can still remain at a relatively higher level.

### 3.3. Temperature-Accelerated Ageing of XLPE

**Figure 4:**
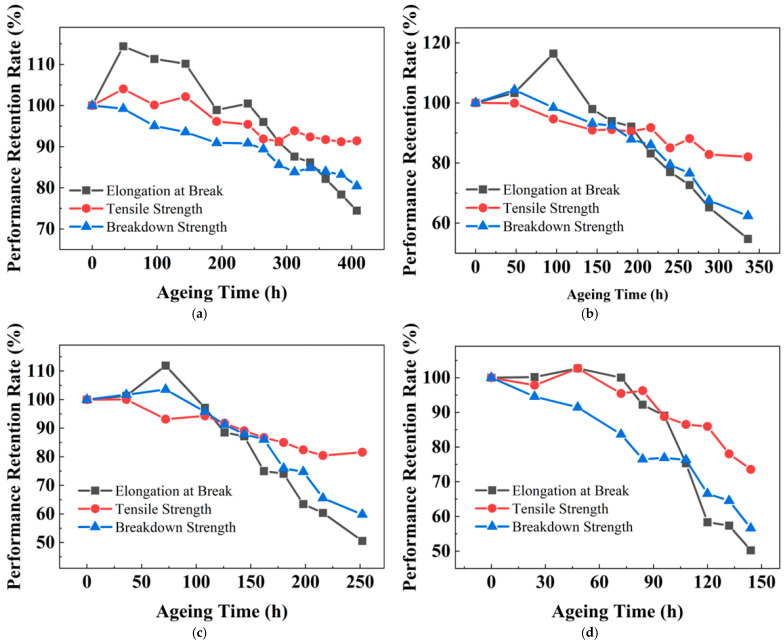
Evolution of various properties retention rates of XLPE during ageing at different ageing temperatures: (**a**) 127.5 °C; (**b**) 135 °C; (**c**) 142.5 °C; and (**d**) 150 °C.

The initial elongation at break, tensile strength, and characteristic breakdown strength of the XLPE are 870.26%, 21.97 MPa, and 550.48 kV/mm, respectively. According to the initial performance results and applying (2), the performance retention rate of XLPE can be obtained. Figure 4 shows the evolution of the performance retention rate during ageing at different temperatures. The results show that, in the early stages of ageing, the elongation at break exhibits a slight increase, potentially due to post-crystallization or structural relaxation. Breakdown strength shows a steady decline or is sometimes maintained at first, then starts to decline. However, the decline of breakdown strength is later surpassed by that of elongation at break. Tensile strength variation is more complex and temperature-dependent. As ageing continues, all properties show a steady decline. At lower ageing temperatures, the decline in mechanical properties exhibits an oscillatory pattern. The evolution of mechanical performance and breakdown performance indicates that ageing has a significant impact on XLPE, and is intensified with the increase in ageing temperature. In addition, the irregular variation in each parameter during the early stages of ageing suggests that the ageing of XLPE during this period is random and complex. The most sensitive parameter, elongation at break, is selected for life assessment. According to standard GBT 11026.2-2012, the end-of-life criterion is defined as a 50% reduction in the initial elongation at break [23]. To improve the accuracy of subsequent fitting, the sampling points were further refined at the stage when the elongation at break returned to the initial level and began to decline steadily, thus ensuring the collection of more reliable data.

**Figure 5:**
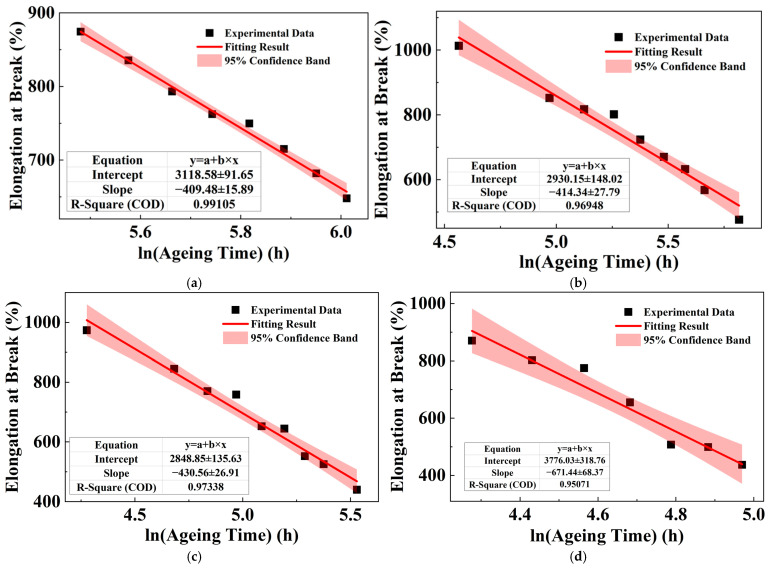
Relationship between XLPE elongation at break and ageing time at different ageing temperatures: (**a**) 127.5 °C; (**b**) 135 °C; (**c**) 142.5 °C; and (**d**) 150 °C.

As discussed in Section 3.1, the degradation of the material’s properties should exhibit a logarithmic dependence on ageing time. Therefore, the sample lifetime can be estimated by fitting this model to the experimental data and extrapolating to the failure criterion [24]. The steady decline in the elongation at break during the late stages of ageing at different temperatures is fitted accordingly. Table 2 shows the fitting equations between the elongation at break of XLPE samples and the logarithmic ageing time, along with the coefficient of determination. In the fitting equations in Table 2, *y* is the elongation at break, with the unit of %; t is the ageing time, with the unit of hours (h). The linear fits, depicted by the red lines in Figure 5, exhibit varied degrees of agreement with the experimental data (black scatters) at different ageing temperatures. Specifically, the fitting result at 127.5 °C shows the highest coefficient of determination (R^2^ = 0.9911), demonstrating excellent consistency.

The ageing lifetime corresponding to a 50% reduction in the initial elongation at break value under different ageing temperatures conditions can be calculated by further extrapolating the fitting equations in Table 2. The results are summarized in Table 3.

By substituting the fitted ageing lifetimes from Table 2 and the corresponding ageing temperatures into Equation (8), respectively, the Arrhenius relationship for the thermo-oxidative ageing of XLPE can be obtained, as Equation (9) shows. The resulting fitting equation exhibits a strong linear correlation, with a coefficient of determination of 0.9925.

(9) $$\ln\left(t_{sample\text{-}XLPE}\right)=11627.58\times \left(1/T\right)-22.45$$

Finally, by extrapolating Equation (9), the expected thermo-oxidative ageing lifetime of XLPE at the operating temperature of 90 °C, *t_sample-XLPE_*, is calculated to be approximately 1.63 (95% confidence interval: 0.59~4.50) years. The SE of ln(*t_sample-XLPE_*) is approximately 0.236 in Supplementary Materials.

### 3.4. Temperature-Accelerated Ageing of PPG

**Figure 6:**
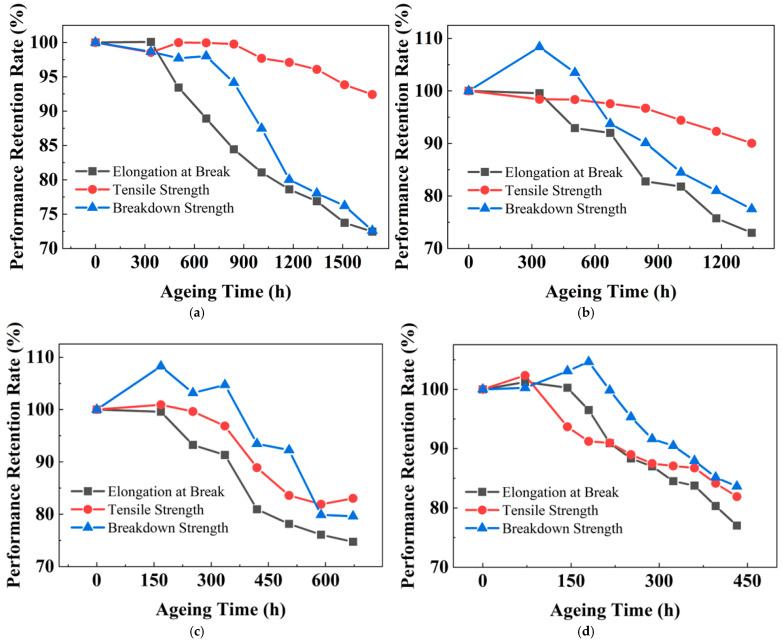
Relationship between various properties of PPG and ageing time under different ageing temperatures: (**a**) 127.5 °C; (**b**) 135 °C; (**c**) 142.5 °C; and (**d**) 150 °C.

The degradation of PPG under thermo-oxidative conditions was monitored through the evolution of elongation at break, tensile strength, and breakdown strength. The initial values are 897.10%, 31.03 MPa, and 592.69 kV/mm, respectively. Figure 6 depicts the retention rates of these properties during ageing at temperatures from 127.5 °C to 150 °C. A distinct pattern emerges for PPG compared to XLPE. The elongation at break, identified as the most sensitive indicator, typically maintains its initial value during early ageing before entering a steady decline stage. Only at 150 °C does a slight increase in the elongation at break occur. As ageing continues, the elongation at break begins to decline steadily. Similar to XLPE, the tensile strength of PPG shows less obvious changes than that in elongation at break. At most temperatures the breakdown strength exhibits a modest initial increase followed by a gradual decrease. Therefore, the end-of-life criterion for PPG is similarly defined as a 50% reduction in the initial elongation at break.

**Figure 7:**
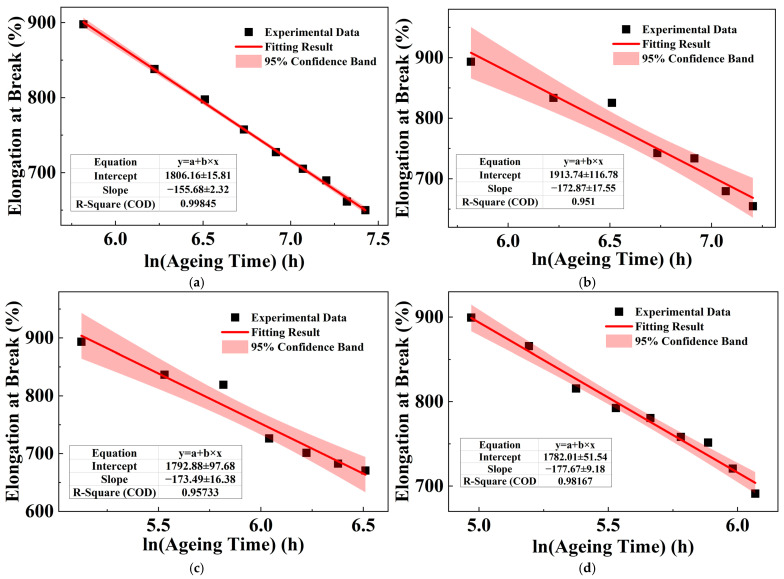
Relationship between elongation at break of PPG and ageing time under different ageing temperatures: (**a**) 127.5 °C; (**b**) 135 °C; (**c**) 142.5 °C; and (**d**) 150 °C.

Table 4 shows the fitting equations between the elongation at break of the PPG sample and the logarithmic ageing time, along with the coefficient of determination during the stable decline stage of elongation at break at different ageing temperatures. In the fitting equations of Table 4, *y* is the elongation at break, with the unit of %; *t* is the ageing time, with the unit of hours (h). Figure 7 shows the relationship between elongation at break and ageing time, where the black scattered points represent the experimental data at different ageing temperatures, and the red line is the fitting result of the elongation at break and the logarithmic ageing time.

By extrapolating the fitting equations in Table 4, the ageing lifetime corresponding to the point when the PPG elongation at break decreases to 50% of its initial value under different ageing temperatures can be calculated. The specific calculation results are summarized in Table 5.

By substituting the ageing lifetimes obtained from the fitting in Table 4, along with the corresponding ageing temperatures, into Equation (8), the fitting equation for the thermo-oxidative ageing lifetime of PPG, is obtained, as shown in Equation (10), with a coefficient of determination of 0.9532:

(10) $$\ln\left(t_{sample\text{-}PPG}\right)=9882.04\times \left(1/T\right)-15.89$$

According to the extrapolation of Equation (10), the expected thermo-oxidative ageing lifetime of PPG at its maximum operating temperature of 110 °C is calculated to be approximately 2.28 (95% confidence interval: 0.65~7.97) years. The SE of ln(*t_sample-PPG_*) is approximately 0.291 in Supplementary Materials.

### 3.5. Thickness-Accelerated Ageing of XLPE

According to the research in Section 3.3, the lifespan of 1.63 years for thin XLPE films derived from temperature acceleration contrasts with the decades-long service expected of full-scale cables. However, the Arrhenius equation used in this study for calculating the thermo-oxidative ageing life of materials is well-established and has been widely applied, with similar discrepancies between theoretical calculations and practical applications also reported [24]. This indicates that the temperature-accelerated ageing experiment designed in this paper is theoretically grounded and reproducible. The discrepancy likely arises from other variables that have not been fully considered. In reality, although cables do not operate in a vacuum environment, the insulation layer is of finite thickness, meaning that diffusion is the primary means by which oxygen reaches the interior of the insulation. This discrepancy is well-documented and often attributed to diffusion-limited oxidation (DLO) [25,26], where the finite oxygen supply to a thick insulation’s interior slows the overall degradation rate. To investigate this phenomenon, ageing experiments were designed using samples with different thicknesses to simulate the influence of thickness on thermo-oxidative ageing, and to better align the cable’s ageing life with practical operational conditions, where oxygen is unevenly distributed from the surface to the interior of the insulation.

**Figure 8:**
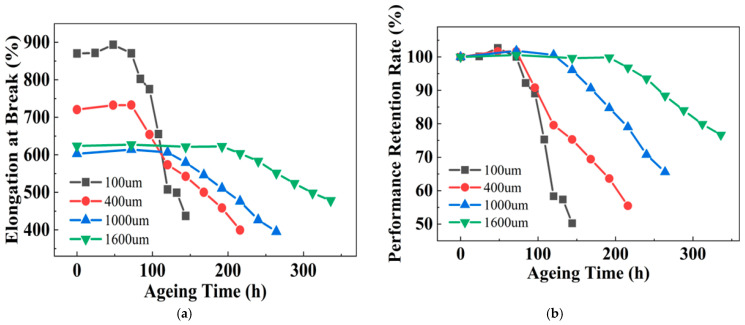
Evolution of elongation at break of XLPE with different thicknesses during ageing: (**a**) experimental data; and (**b**) retention rate.

The research in Section 3.3 has already concluded that the elongation at break of XLPE is the most sensitive parameter to ageing. Therefore, the focus in the subsequent ageing experiments with varying thicknesses is placed on the changes in elongation at break. Figure 8a shows the evolution of the elongation at break of XLPE samples with different thicknesses as a function of ageing time at 150 °C. The elongation at break of all samples increases or maintains the initial level during the early stages of ageing, but as ageing progresses, it begins to decrease gradually. To quantitatively analyze this variation, the same data processing method as in Section 3.1 was adopted. Namely, the initial elongation at break of the samples was taken as the reference, and the elongation at break retention rates for XLPE samples at different ageing stages were then calculated, as shown in Figure 8b.

**Figure 9:**
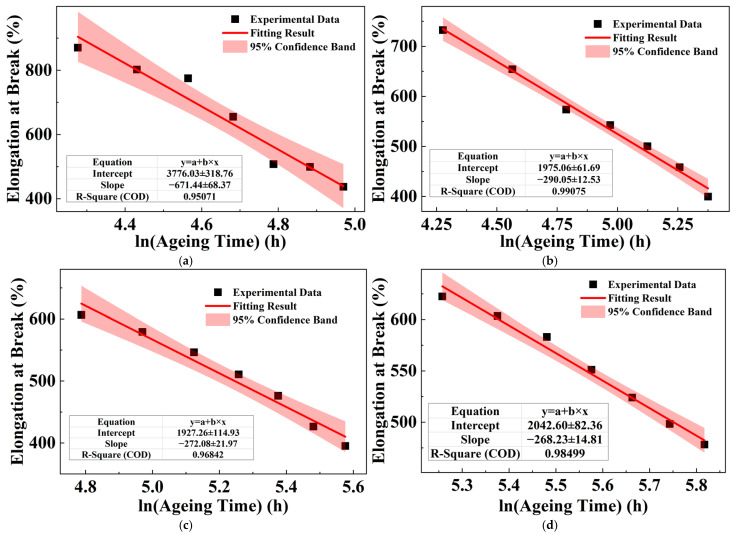
Relationship between elongation at break of XLPE with different thickness and ageing time: (**a**) 100 μm; (**b**) 400 μm; (**c**) 1000 μm; and (**d**) 1600 μm.

The linear fitting parameters for the steady decline in elongation at break against the logarithm of ageing time are summarized in Table 6 of XLPE samples with different thicknesses. In the fitting equations, *y* is the elongation at break, with the unit of %; *t* is the ageing life, with the unit of hours (h). The corresponding experimental data and fitting curves are presented in Figure 9, where the black scatter points indicate the measured values for each thickness, and the red lines depict the respective logarithmic fits.

Extrapolating the fitting equations in Table 6, the ageing lifetime corresponding to the point when the elongation at break of XLPE samples with different thicknesses decreases to 50% of its initial value can be calculated. The calculated lifetimes are summarized in Table 7.

**Figure 10:**
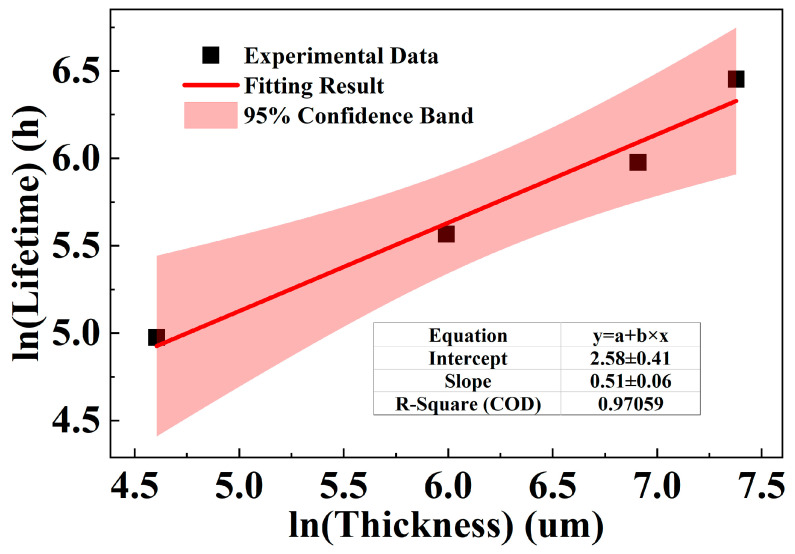
Relationship between ln(thickness) and ln(ageing time) of XLPE.

The results from Table 7 are presented in a double-logarithmic plot, as shown in Figure 10, revealing a clear linear correlation between ln(thickness) and ln(lifetime) for XLPE.

The specific fitting result is shown in Equation (11),

(11) $$\ln\left(t\right)=0.51\times \ln\left(z\right)+2.60$$

where, *t* is the ageing lifetime, with the unit of hours (h), and *z* is the sample thickness parameter, with the unit of µm. The SE of the slope, referred to as *b* in Equation (12), $SE\left(b_{XLPE}\right)$, is approximately 0.062223. The fit shows a strong correlation, with a coefficient of determination of 0.9706. This scaling equation allows the ageing lifetime of XLPE at any thickness to be related to that of a 100 µm reference sample. According to the model, the scaling factor *K* can be expressed as:

(12) $$K=\frac{t_{cable}}{t_{sample}}=e^{\left(b\times \ln z_{cable}-b\times \ln z_{sample}\right)}={\left(\frac{z_{cable}}{z_{sample}}\right)}^{b}$$

To translate the laboratory result to a real cable, a key geometric distinction must be considered. In this experiment, the test samples were suspended in the test chamber, with all external surfaces fully exposed to the air, enabling oxygen to diffuse from both sides. However, in actual cables, the insulation layer adheres to the conductor on one side, leaving only the outer surface exposed to the air. Therefore, for a cable insulation layer with a thickness of 25 mm, the thickness parameter *z_cable_* in Equation (12) should be taken as 50 mm. Thus, ln(*K_XLPE_*) is approximately 3.142, with an SE of it, $SE\left(lnK_{XLPE}\right)$ 0.387, which can be calculated following Equation (13):

(13) $$\mathrm{SE}\left(\ln K_{XLPE}\right)=\ln\left(500\right)\times {\mathrm{SE}}_{b_{XLPE}}\approx 0.3867$$

### 3.6. Thickness-Accelerated Ageing of PPG

**Figure 11:**
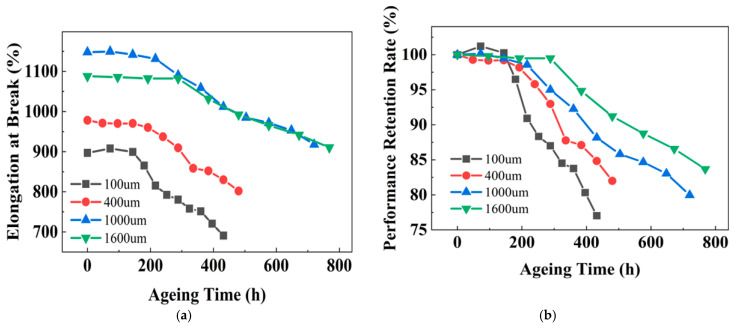
Evolution of elongation at break of PPG with different thicknesses during ageing: (**a**) experimental data; and (**b**) retention rate.

From Section 3.4, it is also concluded that the elongation at break is the most sensitive parameter to ageing for PPG. Therefore, the subsequent thickness-accelerated ageing study focuses on monitoring the evolution in elongation at break. Figure 11a shows the evolution of the elongation at break of PPG samples with different thicknesses over ageing time. Initially, the property remains stable across all thicknesses before entering a steady decline. Using Equation (2), the corresponding retention rates are calculated and presented in Figure 11b.

**Figure 12:**
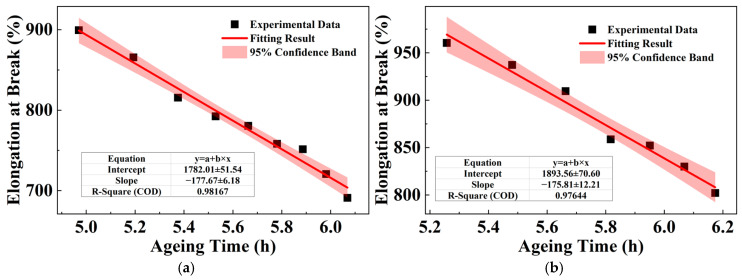

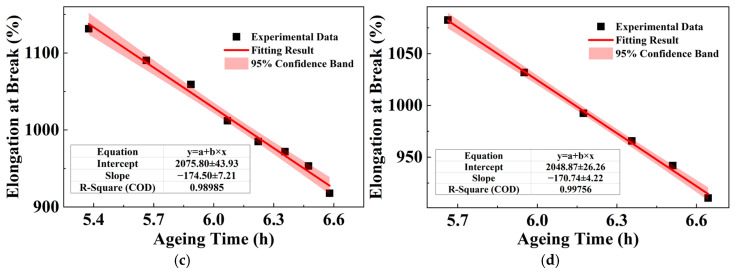
Relationship between the elongation at break in PPG of different thickness and ageing time: (**a**) 100 μm; (**b**) 400 μm; (**c**) 1000 μm; and (**d**) 1600 μm.

The fitting parameters for the steady decline in elongation at break against the logarithm of ageing time are summarized in Table 8 for PPG samples of different thicknesses. In the fitting equations, *y* is the elongation at break, with the unit of %; *t* is the ageing lifetime, with the unit of hours (h). Figure 12 shows the relationship between this elongation at break and ageing time, where the black scattered points represent the experimental data, and the red line represents the fitting result.

By further extrapolating the fitting equations in Table 8, the ageing lifetime corresponding to the point when the elongation at break of PPG samples with different thicknesses reaches 50% of their initial value, can be obtained, as shown in Table 9.

**Figure 13:**
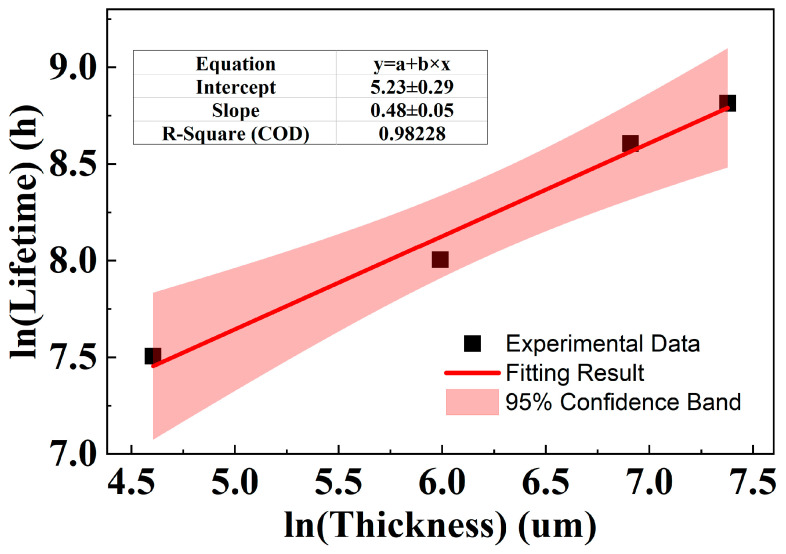
Relationship between ln(thickness) and ln(ageing time) of PPG.

These results from Table 9 are presented in a double-logarithmic plot, shown in Figure 13, which reveals a well-defined linear relationship between ln(thickness) and ln(lifetime) for PPG.

The relationship between thickness and lifetime for PPG is defined by Equation (14),

(14) $$\ln\left(t\right)=0.48\times \ln\left(z\right)+5.25$$

where, *t* is the ageing life, with the unit of h; *z* is the sample thickness parameter, with the unit of μm. The SE of the slope, $SE\left(b_{PPG}\right)$, is 0.04579. The fit shows excellent agreement with the data, yielding a coefficient of determination of 0.9817. Thus, ln(*K_PPG_*) is approximately 2.996, with an SE, $SE\left(lnK_{PPG}\right)$, of 0.029, which can also be calculated following Equation (13). 

### 3.7. Uncertainty Analysis

Considering the influence of sample thickness [27,28,29], the service life of a cable operating long-term at a relatively lower temperature can be derived from the thermo-oxidative ageing lifetime of a 100 µm sample tested at elevated temperatures, as expressed by the following equation:

(15) $$t_{cable}=t_{sample}\times K$$

Since service life cannot be a negative number, it should conform to a log-error propagation model. Consequently, on the logarithmic scale, an additive model is obtained:

(16) $$\ln\left(t_{cable}\right)=\ln\left(t_{sample}\right)\times \ln\left(K\right)$$

For XLPE,

(1) The median result from the temperature-accelerated experiment, $ln(t_{sample-XLPE})$, is approximately 0.489, with a standard error $SE\left[ln\left(t_{sample-XLPE}\right)\right]\approx 0.236$ and degrees of freedom $d_{sample-XLPE}=n-2=2$.
(2) The median result from the thickness-accelerated experiment, $ln(K_{XLPE})$, is approximately 3.142, with a standard error $SE\left[ln\left(K_{XLPE}\right)\right]\approx 0.387,$ and degrees of freedom $d_{K-XLPE}=n-2=2$.

For PPG,

(1) The median result from the temperature-accelerated experiment, $ln(t_{sample-PPG})$, is approximately 0.824, with a standard error $SE\left[ln\left(t_{sample-PPG}\right)\right]\approx 0.291$ and degrees of freedom $d_{sample-PPG}=n-2=2$.
(2) The median result from the thickness-accelerated experiment, $ln(K_{PPG})$, is approximately 2.996, with a standard error $SE\left[ln\left(K_{PPG}\right)\right]\approx 0.029,$ and degrees of freedom $d_{K-XLPE}=n-2=2$.

Given that the two components are independent and both parameters have a sample size of *n* = 4, the t-distribution is employed to construct the median and confidence interval for the lifetime. Therefore, the median of the logarithm of the cable lifetime is $ln\left(t_{cable}\right)=ln\left(t_{sample}\right)+ln\left(K\right)$. Its standard error is calculated as $SE\left[ln\left(t_{cable}\right)\right]=\sqrt{{\left\{SE\left[ln\left(t_{sample}\right)\right]\right\}}^{2}+{\left\{SE\left[ln\left(K\right)\right]\right\}}^{2}}$, with degrees of freedom $d_{cable}=min\left(d_{sample}+d_{K}\right)=2$. When d=2 and α=0.05, the t-table gives a critical value $t_{0.025,2}=4.303$. The 95% confidence interval on the logarithmic scale is given by:

(17) $${\mathrm{CI}}_{\ln}=\ln\left(t_{cable}\right)\pm t_{0.025,2}\times \mathrm{SE}\left(\ln t_{cable}\right)$$

Based on this formula, the median predicted service life for XLPE is 37.75 years, with a 95% confidence interval of [5.37, 265.60] years. For PPG, the median predicted service life is 45.60 years, with a 95% confidence interval of [12.96, 160.45] years.

This relatively wide interval objectively reflects the substantial statistical uncertainty inherent in making long-range extrapolations based on limited accelerated ageing data. This arises from three primary statistical reasons:

(1) Limited sample size in accelerated tests: The temperature acceleration and the thickness-scaling model are each derived from only four accelerated ageing data points. In such small-sample scenarios, quantifying the prediction uncertainty correctly requires the use of the t-distribution with low degrees of freedom, which has heavier tails than the normal distribution, and leading to wider and more conservative confidence intervals. Using a normal distribution would narrow the intervals by over 50%, while significantly underestimating the true uncertainty.
(2) Compounding of uncertainties in a two-step extrapolation: The uncertainties from each step propagate multiplicatively. This compounding effect is a fundamental characteristic of such predictive models and is not an error of the analysis.
(3) Inherent challenge of long-term prediction from short-term tests: Predicting a lifetime of decades based on experiments lasting months or weeks is intrinsically uncertain. The wide confidence interval honestly reflects this challenge.

Therefore, the reported wide confidence intervals do not indicate a flaw in the methodology but rather provide a conservative quantification of the prediction uncertainty. Despite the breadth of the interval, the median prediction retains a significant reference value. 

Based on the above experimental investigations and analyses, the comparisons of key performance between XLPE and PPG are summarized in Table 10. The results confirm that PPG exhibits promising potential for application as a next-generation eco-friendly cable insulation material.

### 3.8. Discussion

The foregoing experimental results demonstrate distinct thermo-oxidative ageing behaviours between XLPE and PPG. PPG not only operates reliably at a higher temperature (110 °C) but also exhibits a substantially longer extrapolated lifespan than XLPE. This enhanced durability necessitates a deeper examination of the underlying chemical and physical degradation mechanisms.

**Figure 14:**
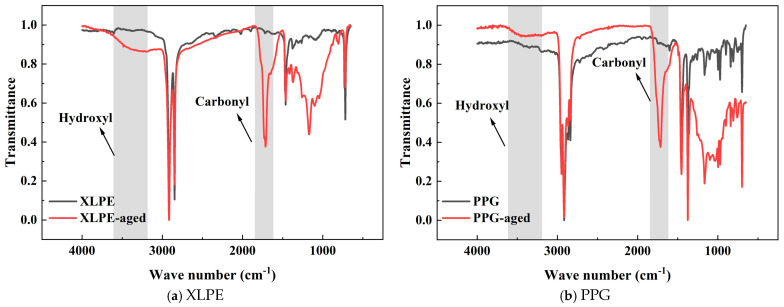
FTIR spectra of XLPE and PPG samples before and after ageing.

The FT-IR spectra of XLPE and PPG samples before and after ageing are obtained, as shown in Figure 14, providing direct evidence of the chemical transformations during ageing.

**Figure 15:**
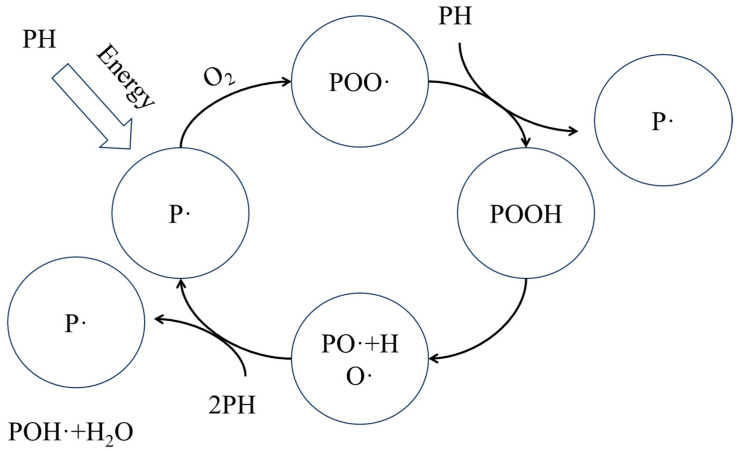
Schematic of the thermo-oxidative ageing reaction pathway for polyolefins.

After ageing, both XLPE and PPG show broadened absorption peaks in the 3200–3700 cm^−1^ region, which meet the characteristics of the infrared absorption peaks of hydroxyl (O-H) stretching, suggesting that a large number of hydroxyl groups form during the ageing process. In addition, both samples exhibit obvious absorption peaks near 1720 cm^−1^, indicating that a large number of carbonyl (C=O) groups are generated after ageing. A decrease in transmittance around 1175 cm^−1^ (C-O stretching) is also observed. According to the chain reaction of ageing, as Figure 15 shows, the appearance of carbonyl groups and hydroxyl groups both illustrates the ageing behaviour of XLPE and PPG molecules. For XLPE, main chain scission and branching, formation of oxides, and destruction or formation of new cross-linked structures occur, which disrupt the molecular regularity, broadening the peak shape. For PP, the degradation and oxidation of methyl side groups, the scission of main chain C-C bonds, and a decrease in crystallinity occur. The destruction of molecular structure and change in functional groups caused by the mentioned processes during ageing superimpose on each other, ultimately leading to an overall decrease in transmittance in the range of 1335~855 cm^−1^. Although compared with XLPE, there are a lot of C–H bonds possessing lower bond energy on the PP macromolecular chains. And thus, they are easier to scission during ageing. The grafting modification consumes these vulnerable sites by cleaving them and attaching benzene rings. This effectively reduces the number of these weak linkages on the main PP chains, thereby lowering their probability of scission.

**Figure 16:**
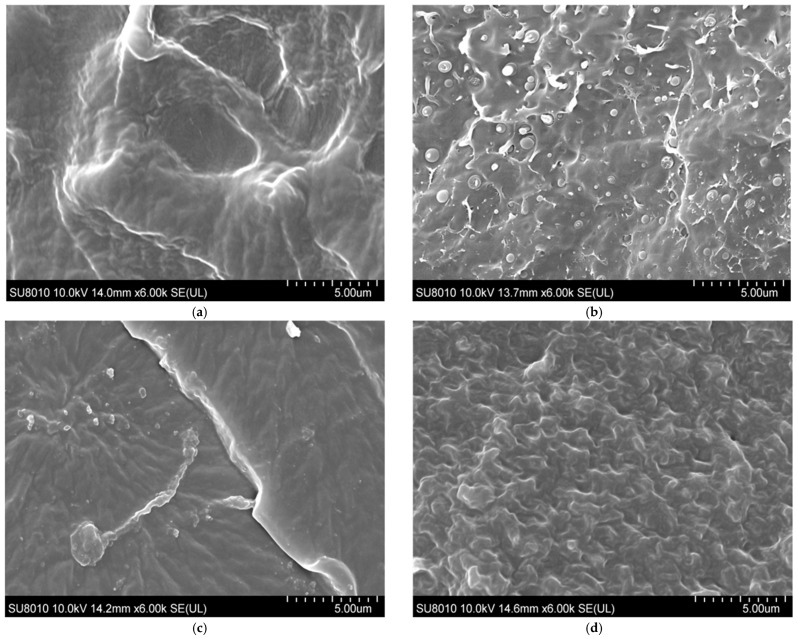
SEM images of fracture surfaces: (**a**) unaged XLPE; (**b**) unaged PPG; (**c**) aged XLPE; and (**d**) aged PPG.

The superior ageing resistance of PPG, as quantified in the lifespan predictions, can be attributed to its modified microstructure. The SEM images are shown in Figure 16, revealing the microstructure of the materials. The dense spherical features in unaged PPG are aggregations of grafted monomers. After ageing, these features become less distinct and appear more integrated with the PP matrix. It is likely that the aggregations of the grafted small molecules may degrade earlier, potentially delaying the degradation process of PP macromolecular chains and contributing to PPG’s enhanced ageing resistance.

**Figure 17:**
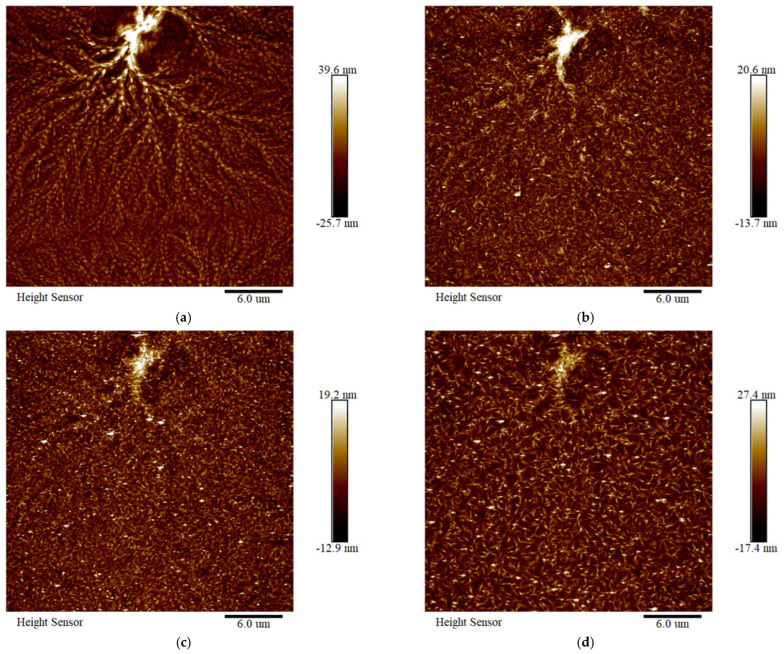
In situ ageing AFM images of XLPE: (**a**) unaged XLPE; (**b**) XLPE aged for 3 h; (**c**) XLPE aged for 6 h; and (**d**) XLPE aged for 9 h.

In situ ageing observation via AFM provides a clearer visualization of the degradation processes in both XLPE and PPG samples. When aged at the same temperature, the XLPE sample exhibits more rapid dissolution of its spherulites as Figure 17a,b show. Potential re-crosslinking may occur at the original spherulite boundary, leading to a narrowing of the boundary. As ageing continued, the initially formed spherulites further dissolved, exposing the underlying cross-linked network of XLPE. The height and dimensions of crystalline nuclei decrease, indicating the disentanglement of molecular chains that originally contributed to strong interactions. This microstructural deterioration corresponds to the decline in macroscopic properties.

**Figure 18:**
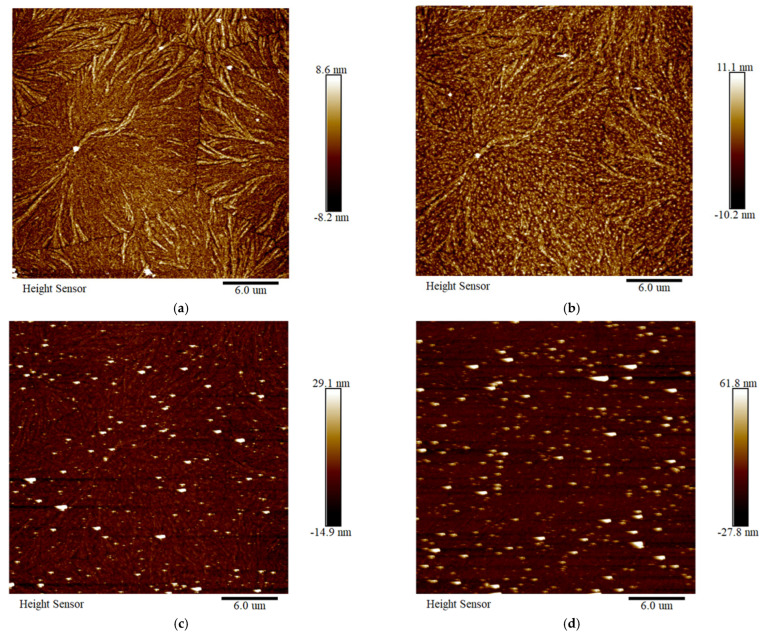
In situ ageing AFM images of PPG: (**a**) unaged PPG; (**b**) PPG aged for 6 h; (**c**) PPG aged for 27 h; and (**d**) PPG aged for 36 h.

Figure 18 shows the in situ ageing process of the PPG sample, revealing more complex ageing behaviour. In the first stage, the spherulitic structure becomes more perfected under thermal influence. The size of the aggregated grafted nodules decreases as indicated by the middle red circle in Figure 18b, while aggregation of other grafted monomers is observed, as shown in Figure 18b in the left red circle. These reaggregated monomers enhance the interactions between the grafted PP molecular chains, and the more perfected crystalline structure improves the stability of the spherulites. This explains the slight improvement or maintenance of macroscopic properties during the initial ageing period. With further ageing, the sample surface becomes inevitably contaminated by airborne dust, yet the aggregated monomers continue to shrink in size while the overall spherulitic morphology remains largely intact. In later stages, due to increased surface contamination and extensive melting of the spherulites, identifying detailed structural features becomes challenging.

## 4. Conclusions

Temperature-accelerated ageing experiments demonstrate that elongation at break is a reliable parameter for predicting the service life of both cross-linked polyethylene (XLPE) and grafted polypropylene (PPG) insulation materials. Based on the Arrhenius equation, the thermo-oxidative ageing lifetime of a 100 μm thick XLPE sample operating at 90 °C and a PPG sample operating at 110 °C is estimated to be approximately 1.63 and 2.28 years, respectively. These calculated results are significantly lower than the lifespan observed in the practical application of widely used XLPE cables, implying that certain experimental limitations or unaccounted factors in the accelerated ageing model may contribute to this discrepancy. To address the gap between laboratory tests and engineering applications, a “thickness–lifetime” scaling relationship is established, and a life prediction function t = f(T, z) is proposed, which integrates the Arrhenius temperature dependence with a power-law thickness dependence. This function provides a more economical and rapid methodology for the lifespan prediction of cable insulation materials in engineering practice.

The results confirm the superior thermo-oxidative ageing resistance of PPG, especially when operating at its higher maximum service temperature of 110 °C. Its longer estimated cable lifespan, combined with its excellent recyclability, underscores PPG’s significant potential as a next-generation insulation material for high-voltage cables, which is capable of meeting the demands of future power capacity expansion.

## Figures and Tables

**Table 1:** Initial tensile properties of XLPE and PPG with different thicknesses.

Sample	Thickness (μm)	Elongation at Break (%)	Tensile Strength (MPa)	
XLPE	100	870.26 (±103.44)	21.97 (±1.38)	
	400	720.51 (±52.99)	20.01 (±0.67)	
	1000	602.85 (±106.53)	19.81 (±1.25)	
	1600	623.53 (±65.1)	20.54 (±0.78)	
PPG	100	897.10 (±92.64)	31.03 (±1.52)	
	400	978.29 (±108.70)	31.08 (±1.83)	
	1000	1147.91 (±119.71)	29.29 (±1.14)	
	1600	1087.99 (±75.87)	37.19 (±0.85)	

**Table 2:** Fitting parameters for XLPE elongation at break vs. ln(ageing time).

Ageing Temperatures (°C)	Fitting Equation	Standard Error (SE) of Slope	SE of Intercept	Coefficient of Determination
127.5	*y* = −409.48 × ln(*t*) + 3118.58	15.89	91.65	0.9911
135	*y* = −414.34 × ln(*t*) + 2930.15	27.79	148.02	0.9695
142.5	*y* = −430.56 × ln(*t*) + 2848.85	26.91	135.63	0.9734
150	*y* = −671.44 × ln(*t*) + 3776.03	68.37	318.76	0.9507

**Table 3:** Calculated lifetimes of XLPE (100 µm) at different ageing temperatures.

Ageing Temperatures (°C)	Initial Elongation at Break (%)	Lifetime (h)
127.5	870.26	701.56
135		412.27
142.5		272.05
150		144.85

**Table 4:** Fitting parameters for PPG elongation at break vs. ln(ageing time).

Ageing Temperatures (°C)	Fitting Equation	SE of Slope	SE of Intercept	Coefficient of Determination
127.5	*y* = −155.68 × ln(*t*) + 1806.16	2.32	15.81	0.9985
135	*y* = −172.87 × ln(*t*) + 1913.74	17.55	116.78	0.951
142.5	*y* = −173.49 × ln(*t*) + 1792.88	16.38	97.68	0.9573
150	*y* = −177.67 × ln(*t*) + 1782.01	9.18	51.54	0.9817

**Table 5:** Calculated lifetimes of PPG (100 µm) at different ageing temperatures.

Ageing Temperatures (°C)	Initial Value of Elongation at Break (%)	Lifetime (h)
127.5	897.10	6127.34
135		4796.66
142.5		2318.66
150		1817.58

**Table 6:** Fitting parameters for XLPE elongation at break (different thicknesses) vs. ln(ageing time) at 150 °C.

Thickness (μm)	Fitting Equations	SE of Slope	SE of Intercept	Coefficient of Determination
100	*y* = −671.44 × ln(*t*) + 3776.03	68.37	82.36	0.9507
400	*y* = −290.05 × ln(*t*) + 1975.07	12.53	114.93	0.9908
1000	*y* = −272.08 × ln(*t*) + 1927.26	21.97	61.69	0.9684
1600	*y* = −268.23 × ln(*t*) + 2045.60	14.81	318.76	0.9850

**Table 7:** Calculated lifetimes of XLPE with different thicknesses.

Thickness (μm)	Initial Value of Elongation at Break (%)	Lifetime (h)
100	870.26	144.85
400	720.51	261.74
1000	602.85	393.95
1600	623.53	634.53

**Table 8:** Fitting parameters for PPG elongation at break (different thicknesses) vs. ln(ageing time) at 150 °C.

Thickness (μm)	Fitting Equations	SE of Slope	SE of Intercept	Coefficient of Determination
100	*y* = −177.67 × ln(*t*) + 1782.01	6.18	51.54	0.9817
400	*y* = −175.13 × ln(*t*) + 1891.04	12.21	70.60	0.9839
1000	*y* = −174.50 × ln(*t*) + 2075.80	7.21	43.93	0.9899
1600	*y* = −170.74 × ln(*t*) + 2048.87	4.22	26.26	0.9976

**Table 9:** Calculated lifetimes of PPG with different thicknesses.

Thickness (μm)	Initial Value of Elongation at Break (%)	Lifetime (h)
100	897.10	1825.89
400	978.29	2995.57
1000	1147.91	5467.39
1600	1087.99	6726.68

**Table 10:** Comparisons of XLPE and PPG.

Properties	XLPE	PPG
Maximum operating temperature (°C)	90	110
*T_m_* (°C)	102	159
*T_onset_* (°C)	435	359
Conductivity at 30 °C under 20 kV/mm (S/m)	2.23 × 10^−15^	1.90 × 10^−16^
Breakdown Strength at 30 °C (kV/mm)	550.48	592.69
Elongation at break of a 100 μm sample	870.26	897.10
Expected lifespan (Median value) (years)	37.5	45.60

## Data Availability

The original contributions presented in this study are included in the article/Supplementary Material. Further inquiries can be directed to the corresponding author.

## Supplementary Materials

The following supporting information can be downloaded at: https://www.mdpi.com/article/10.3390/polym18030386/s1.
