# Supplementary material for: Comparisons of Thermo-Oxidative Ageing Performance and Lifespan Evaluation of Grafted Polypropylene and XLPE Cables: Combined Effect of Temperature and Thickness
Source: Polymers (Basel). 2026 Jan 31;18(3):386. doi: 10.3390/polym18030386 (PMC12899345; doi:10.3390/polym18030386)
Supplement: Supplementary file 1 [file polymers-18-00386-s001.zip › polymers-4112801-supplementary.pdf]

FOR XLPE:

SE of ln(t) of Equation (9) is 0.236 approximately. ln(t) is 0.489.

For Equation (9), the SE of slope of is 717.00945, s (RMSE) is 0.07093. n (number of data points) is 4.

$$T_1 = 127.5℃ = (127.5 + 273.15)\text{K}=400.65\text{K} \rightarrow x_1 = \frac{1}{T_1} = \frac{1}{400.65} \approx 0.0024959$$

$$T_2 = 135℃ = (135 + 273.15)\text{K}=408.15\text{K} \rightarrow x_2 = \frac{1}{T_2} = \frac{1}{408.15} \approx 0.0024501$$

$$T_3 = 142.5℃ = (142.5 + 273.15)\text{K}=415.65\text{K} \rightarrow x_3 = \frac{1}{T_3} = \frac{1}{415.65} \approx 0.0024059$$

$$T_4 = 150℃ = (150 + 273.15)\text{K}=423.15\text{K} \rightarrow x_4 = \frac{1}{T_4} = \frac{1}{423.15} \approx 0.0023632$$

$$T_0 = 90℃ = (90 + 273.15)\text{K}=363.15\text{K} \rightarrow x_0 = \frac{1}{T_0} = \frac{1}{363.15} \approx 0.0027538$$

$$\bar{x} = \frac{0.0024959 + 0.0024501 + 0.0024059 + 0.0023632}{4} \approx 0.0024288$$

$$SS_{xx} = \sum\left(x_i - \bar{x}\right)^2$$

$$\left(x_1 - \bar{x}\right)^2 = (0.0024959 - 0.0024288)^2 = 0.0000671^2 = 4.50\times10^{-9}$$

$$\left(x_2 - \bar{x}\right)^2 = (0.0024501 - 0.0024288)^2 = 0.0000213^2 = 4.54\times10^{-10}$$

$$\left(x_3 - \bar{x}\right)^2 = (0.0024059 - 0.0024288)^2 = (-0.0000229)^2 = 5.24\times10^{-10}$$

$$\left(x_4 - \bar{x}\right)^2 = (0.0023632 - 0.0024288)^2 = (-0.0000656)^2 = 4.30\times10^{-9}$$

$$SS_{xx} = (4.5 + 0.454 + 0.524 + 4.35)\times10^{-9} = 9.778\times10^{-9}$$

$$\text{SE}(y_{90}) = s\times\sqrt{\frac{1}{n} + \frac{\left(x_0 - \bar{x}\right)^2}{SS_{xx}}}$$

$$\frac{1}{n} = \frac{1}{4} = 0.25$$

$$x_0 - \bar{x} = 0.0027538 - 0.0024288 = 0.0003250$$

$$\left(x_0 - \bar{x}\right)^2 = 0.000325^2 = 1.056\times10^{-7}$$

$$\frac{\left(x_0 - \bar{x}\right)^2}{SS_{xx}} = \frac{1.056\times10^{-7}}{9.778\times10^{-9}} \approx 10.80$$

$$\sqrt{\frac{1}{n} + \frac{\left(x_0 - \bar{x}\right)^2}{SS_{xx}}} = \sqrt{0.25 + 10.80} = \sqrt{11.05} \approx 3.324$$

$$\mathrm{SE}\left(y_{90}\right) = s \times \sqrt{\frac{1}{n} + \frac{\left(x_0 - \bar{x}\right)^2}{SS_{xx}}} = 0.07093 \times 3.324 \approx 0.2358$$

$$\ln\left(t_{sample}\right) = \ln\left(1.63\right) \approx 0.489$$

<u>SE of ln(K) of Equation (12) is 0.387 approximately. ln(K) is 3.142.</u>

For Equation (12), the slope is 0.50555, with the SE of the slope of it being 0.06223.

$$\mathrm{SE}\left(\ln K\right) = \mathrm{SE}_b \times \ln\left(500\right) = 6.2146 \times 0.06223 \approx 0.387$$

$$\ln K = b \times \ln\left(500\right) = 0.50555 \times 6.2146 \approx 3.142$$

<u>Median service life of XLPE cable and 95% Confidence Band:</u>

Following Equation (15)

$$\ln\left(t_{cable}\right) = \ln\left(t_{sample}\right) \times \ln\left(K\right) = 0.489 + 3.142 = 3.631$$

95% Confidence Band:

$$\mathrm{SE}\left(\ln t_{cable}\right) = \sqrt{\mathrm{SE}\left(y_{90}\right)^2 + \mathrm{SE}\left(\ln K\right)^2} = \sqrt{0.236^2 + 0.387^2} = \sqrt{0.055696 + 0.149769} \approx \sqrt{0.205465} \approx 0.4533$$

$$\ln\left(t_{cable}\right) \pm t_{0.025,2} \times \mathrm{SE}\left(\ln t_{cable}\right) = 3.631 \pm 4.303 \times 0.4533 = 3.631 \pm 1.951$$

$$t_{cable} = e^{3.631} \approx 37.75$$

$$CI_L = e^{1.680} \approx 5.37$$

$$CI_U = e^{5.582} \approx 265.60$$

FOR PPG:

SE of ln(t) of Equation (10) is 0.291 approximately.

For Equation (10), the SE of slope of is 1548.42727, s (RMSE) is 0.15318. n (number of data points) is 4.

$$T_1 = 127.5℃ = (127.5 + 273.15)\text{K}=400.65\text{K} \rightarrow x_1 = \frac{1}{T_1} = \frac{1}{400.65} \approx 0.0024959$$

$$T_2 = 135℃ = (135 + 273.15)\text{K}=408.15\text{K} \rightarrow x_2 = \frac{1}{T_2} = \frac{1}{408.15} \approx 0.0024501$$

$$T_3 = 142.5℃ = (142.5 + 273.15)\text{K}=415.65\text{K} \rightarrow x_3 = \frac{1}{T_3} = \frac{1}{415.65} \approx 0.0024059$$

$$T_4 = 150℃ = (150 + 273.15)\text{K}=423.15\text{K} \rightarrow x_4 = \frac{1}{T_4} = \frac{1}{423.15} \approx 0.0023632$$

$$T_0 = 110℃ = (110 + 273.15)\text{K}=383.15\text{K} \rightarrow x_0 = \frac{1}{T_0} = \frac{1}{383.15} \approx 0.002610$$

$$\bar{x} = \frac{0.0024959 + 0.0024501 + 0.0024059 + 0.0023632}{4} \approx 0.0024288$$

$$SS_{xx} = \sum\left(x_i - \bar{x}\right)^2$$

$$\left(x_1 - \bar{x}\right)^2 = (0.0024959 - 0.0024288)^2 = 0.0000671^2 = 4.50\times10^{-9}$$

$$\left(x_2 - \bar{x}\right)^2 = (0.0024501 - 0.0024288)^2 = 0.0000213^2 = 4.54\times10^{-10}$$

$$\left(x_3 - \bar{x}\right)^2 = (0.0024059 - 0.0024288)^2 = (-0.0000229)^2 = 5.24\times10^{-10}$$

$$\left(x_4 - \bar{x}\right)^2 = (0.0023632 - 0.0024288)^2 = (-0.0000656)^2 = 4.30\times10^{-9}$$

$$SS_{xx} = (4.5 + 0.454 + 0.524 + 4.35)\times10^{-9} = 9.778\times10^{-9}$$

$$\text{SE}(y_{110}) = s\times\sqrt{\frac{1}{n} + \frac{\left(x_0 - \bar{x}\right)^2}{SS_{xx}}}$$

$$\frac{1}{n} = \frac{1}{4} = 0.25$$

$$x_0 - \bar{x} = 0.002610 - 0.0024288 = 0.0001812$$

$$\left(x_0 - \bar{x}\right)^2 = 0.0001812^2 \approx 3.283\times10^{-8}$$

$$\frac{\left(x_0 - \bar{x}\right)^2}{SS_{xx}} = \frac{3.283\times10^{-8}}{9.778\times10^{-9}} \approx 3.358$$

$$\sqrt{\frac{1}{n} + \frac{\left(x_0 - \bar{x}\right)^2}{SS_{xx}}} = \sqrt{0.25 + 3.358} = \sqrt{3.608} \approx 1.899$$

$$\mathrm{SE}\left(y_{110}\right)=s\times\sqrt{\frac{1}{n}+\frac{\left(x_0-\bar{x}\right)^2}{SS_{xx}}}=0.15318\times1.899\approx0.291$$

$$\ln\left(t_{sample}\right)=\ln\left(2.28\right)\approx0.824$$

<u>SE of ln(K) of Equation (14) is 0.029 approximately. ln(K) is 2.996.</u>

For Equation (14), the slope is 0.48212, with the SE of the slope of it being 0.04579.

$$\mathrm{SE}\left(\ln K\right)=\mathrm{SE}_b\times\ln\left(500\right)=0.04579\times6.2146\approx0.029$$

$$\ln K=b\times\ln\left(500\right)=0.48212\times6.2146\approx2.996$$

<u>Median service life of PPG cable and 95% Confidence Band:</u>

Following Equation (15)

$$\ln\left(t_{cable}\right)=\ln\left(t_{sample}\right)\times\ln\left(K\right)=0.824+2.996=3.820$$

95% Confidence Band:

$$\mathrm{SE}\left(\ln t_{cable}\right)=\sqrt{\mathrm{SE}\left(y_{110}\right)^2+\mathrm{SE}\left(\ln K\right)^2}=\sqrt{0.291^2+0.029^2}=\sqrt{0.084681+0.000841}\approx\sqrt{0.085522}\approx0.2924$$

$$\ln\left(t_{cable}\right)\pm t_{0.025,2}\times\mathrm{SE}\left(\ln t_{cable}\right)=3.820\pm4.303\times0.2924=3.820\pm1.258$$

$$t_{cable}=e^{3.820}\approx45.60$$

$$CI_L=e^{2.562}\approx12.96$$

$$CI_U=e^{5.078}\approx160.45$$
